# Cardiac resynchronization therapy in heart failure patients by using left bundle branch pacing

**DOI:** 10.3389/fcvm.2022.990016

**Published:** 2022-08-23

**Authors:** Ying Gu, Yanming Li, Ying Zhu, Xiuyu Lin, Tian Tian, Qigao Zhang, Jianbin Gong, Lei Wang, Jianhua Li

**Affiliations:** ^1^Department of Cardiology, Jinling Hospital, Nanjing University School of Medicine, Nanjing, China; ^2^Department of Ultrasonic Diagnosis, Jinling Hospital, Nanjing University School of Medicine, Nanjing, China

**Keywords:** left bundle branch pacing, cardiac resynchronization therapy, left bundle branch block, heart failure, pacing threshold

## Abstract

**Background:**

Left bundle branch pacing (LBBP) is emerging as an effective alternative to achieve cardiac resynchronization therapy (CRT) and improve heart function. The purpose of our study was to investigate the feasibility and efficacy of LBBP in heart failure patients with left ventricular ejection fraction (LVEF) <50% and left bundle branch block (LBBB).

**Methods:**

All patients with complete LBBB and LVEF <50% were retrospectively included in the study from April 2018 to April 2021 and underwent CRT *via* LBBP implantation. ECG, pacing parameters, the New York Heart Association (NYHA) functional class, echocardiographic measurements, and complications were recorded and analyzed at implant and during follow-up of 1, 6, and 12 months.

**Results:**

Left bundle branch pacing was successful in all 34 patients (mean age 65.6 ± 11.2 years, 67.6% men). A significant decrease in QRS duration (QRSd) was observed after the LBBP operation for 1 month (153.2 ± 1.7 vs. 111.9 ± 2.6 ms, *p* < 0.01). LBB capture threshold and R-wave amplitude remained stable at 12-month follow-up when compared with implantation values (0.62 ± 0.13 V @ 0.4 ms vs. 0.73 ± 0.21 V @ 0.4 ms, 12.02 ± 5.68 mV vs. 8.58 ± 4.09 mV, respectively). LVEF increased significantly (35.28 ± 1.70% vs. 51.09 ± 1.71%, *p* < 0.01) accompanied with reduced left ventricular end-diastolic dimension (LVEDd; 65.3 ± 1.99 vs. 53.58 ± 2.07 mm, *p* < 0.01) and left atrial dimension (LAD; 49.03 ± 1.32 vs. 40.67 ± 1.58 mm, *p* < 0.01). Normalized LVEF (LVEF ≥ 50%) was found in 70.5% of patients at 12 months. The NYHA classification, brain natriuretic peptide (BNP), and 6-minute walk test (6MWT) were significantly improved at follow-up of 12 months (all *p* < 0.01 vs. baseline). No deaths or heart failure hospitalizations were observed during the follow-up period.

**Conclusion:**

The current work suggested that LBBP was feasible with a high success implantation rate and effective to correct LBBB and improved left ventricular structure and function with a low and stable pacing threshold.

## Introduction

Cardiac resynchronization therapy (CRT) by biventricular pacing (BVP) was widely used to provide clinical benefits in heart failure patients with decreased left ventricular ejection fraction (LVEF) and left bundle branch block (LBBB) (1–3). Though ventricular dyssynchrony and heart failure symptoms could be improved, approximately one-third of the patients had no response to traditional CRT. Compared with BVP, His Bundle Pacing (HBP) might correct LBBB and achieve better ventricular resynchronization and heart functional improvements (4, 5). However, HBP was found to require higher LBBB correction capture thresholds, lower R wave amplitudes, and smaller implant success rates, which limited the widespread application of the HBP technique (6, 7).

As an innovative technique, left bundle branch pacing (LBBP) has emerged to be an alternative method by pacing the left bundle branch bypassing the block region, resulting in physiological pacing and achieving electrical synchrony of the left ventricle. The first case of successful cardiac resynchronization by LBBP was conducted by Huang et al. (8). Increasing evidence showed that LBBP could develop relatively narrow QRS duration (QRSd), fast peak left ventricular activation time (LVAT), and LBBB correction with a low and stable pacing output (9, 10). While the clinical benefits and adverse effects had been described in several case reports and works, the clinical outcome of our center had not been reported.

The aim of the present study was to identify the clinical efficacy and safety of LBBP in heart failure with LBBB.

## Methods

### Study population

This was a retrospective, non-randomized, and single-center study performed between April 2018 and April 2021. Patients who met the following criteria were included (1) ECG with complete LBBB according to Strauss criteria (11); (2) LVEF <50% with heart failure symptoms; and (3) life expectancy >1 year. Patients with an age ≤ 18 years and pregnancy were excluded. The study was performed in accordance with the principles established in the Declaration of Helsinki and approved by the Ethics Committee of Jinling Hospital, Nanjing University School of Medicine. All subjects provided written informed consent.

### Implant procedure

The conduction of LBBP was performed according to the previous reports (12, 13). First, the His bundle was marked using the Select Secure Lead (model 3830, Medtronic, Minneapolis, MN) through the C315 His delivery sheath (Medtronic, Minneapolis, MN). Subsequently, the lead was directed toward the ventricular side 1–2 cm along the line from His site to the right ventricular (RV) apex at right anterior oblique (RAO) 30° and then deeply screwed into the inter-ventricular septum. Once a right bundle branch block (RBBB) morphology was achieved with paced QRSd in lead V1, further lead advancement was stopped. Sti-LVAT was recorded as the interval from the pacing stimulus to the peak of the R wave in leads V4–V6 at high and low outputs (14). Finally, the depth of the lead in the septum was determined by contrast injection through the sheath at the left anterior oblique (LAO) 30°.

### Data collection and follow-up

All enrolled patients were followed in our center at 1-, 6-, and 12 months post-operation. Baseline demographics and medical history were documented at enrollment. Bipolar R-wave amplitude, unipolar LBB capture threshold, and unipolar pacing impedance were collected at implant and follow-up visits. Electrocardiographic and echocardiographic parameters were recorded, such as QRSd, LVEF, left ventricular end-diastolic dimension (LVEDd), left atrial dimension (LAD), degree of mitral regurgitation (MR), and tricuspid regurgitation (TR, mild as first degree, moderate as second degree, and severe as third degree). The measurement of QRSd was achieved from the onset of the intrinsicoid R wave noted in lead V1 or V2 (15). Brain natriuretic peptide (BNP) levels, the New York Heart Association (NYHA) functional class, and the 6-min walk test (6MWT) were measured and compared at baseline and follow-up. Procedure-related complications, data regarding the significant increase of pacing threshold, lead dislodgement and perforation, infections, embolism, stroke, heart failure rehospitalizations, and death were collected during operation and post-operation visits.

### Statistical analysis

Statistical analysis was performed by SPSS version 22.0 software. Shapiro-Wilk test was used to evaluate the normality of the quantitative data. Continuous variables were presented as mean ± standard deviation (SD) or median (interquartile range). The difference between 2 groups was analyzed by paired Student's *t*-test, whereas the difference between multiple groups was analyzed by one-way ANOVA or Kruskal–Wallis *H* test. Categorical variables were presented as percentages. A *p*-value < 0.05 was considered to indicate a statistical significance.

## Results

### Baseline characteristics of heart failure patients

A total of 34 patients was included in the study, who had symptomatic heart failure with decreased LVEF (35.3 ± 9.9%) and LBBB with wide QRSd (153.2 ± 1.7 ms). The average age was 65.6 ± 11.2 years old, and 23 of these patients (67.6%) were men. Six patients had paroxysmal atrial fibrillation (AF), two patients had a first-degree atrioventricular block (AVB), and 7 patients had ischemic cardiomyopathy (ICM). The baseline characteristics of the subjects are shown in Table 1.

**Table 1:** Baseline characteristics.

	**LBBP (*n* = 34)**
Age (years)	65.6 ± 11.2
Male, *n* (%)	23 (67.6)
Hypertension, *n* (%)	21 (61.8)
Diabetes, *n* (%)	9 (26.5)
AF, *n* (%)	6 (17.6)
AV block, *n* (%)	2 (5.9)
ICM, *n* (%)	7 (20.6)
Intrinsic QRS duration (ms)	153.2 ± 6.7
Hb (g/L)	132.6 ± 20.5
Cr (umol/L)	83.2 (61.2, 113.9)
BNP (pmol/L)	385.8 (170.1, 896.6)
Baseline LVEF (%)	35.3 ± 9.9
NYHA functional class
II, *n* (%)	4 (11.8)
III, *n* (%)	18 (52.9)
IV, *n* (%)	12 (35.3)
Medicine history
Beta-blockers, *n* (%)	27 (79.4%)
ACE inhibitors/ARB, *n* (%)	21 (61.8%)
Diuretics, *n* (%)	31 (91.2%)

### ECG and pacing parameters in patients with LBBP implantation

**Figure 1:**
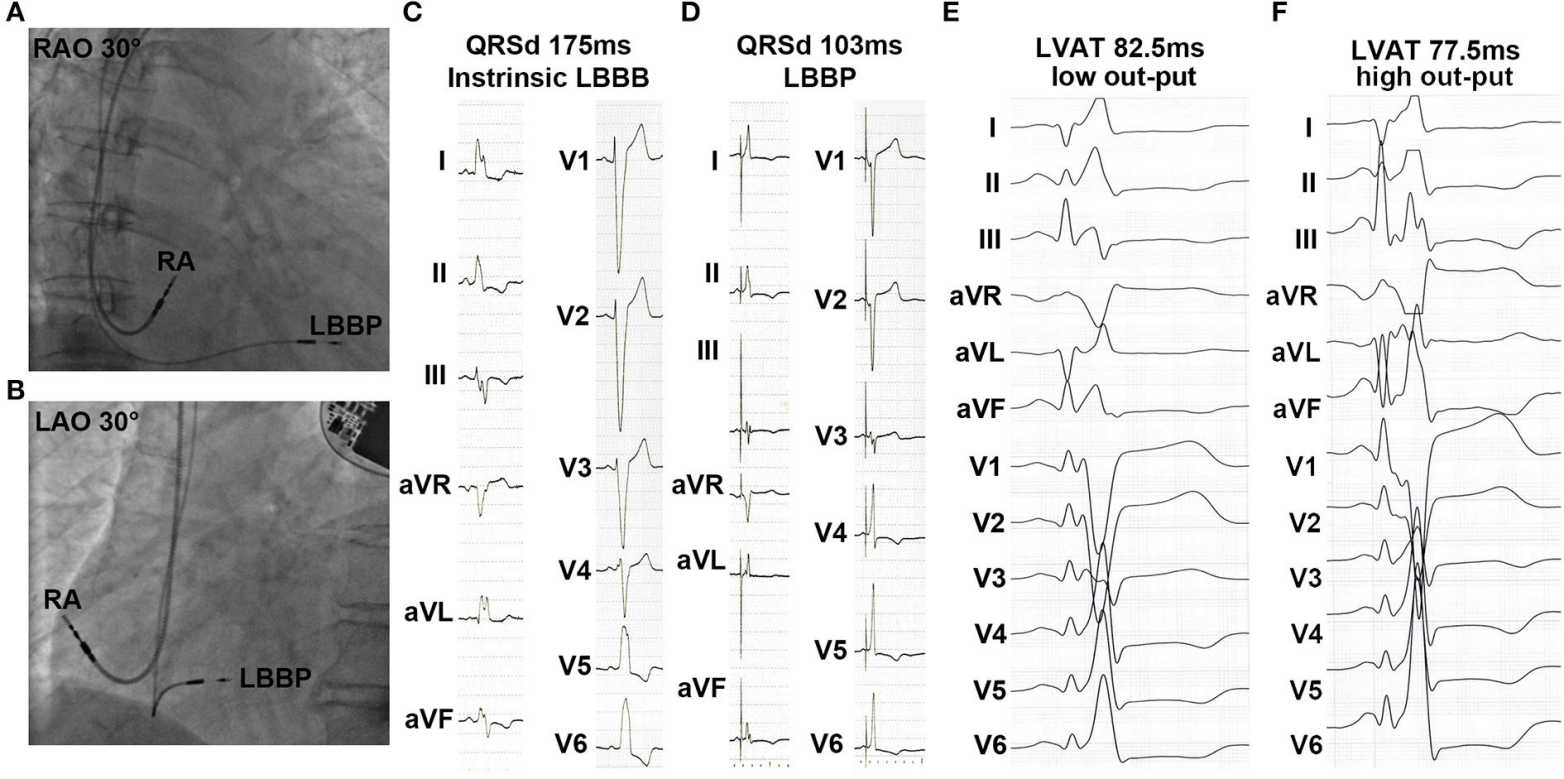
Characteristics of left bundle branch pacing (LBBP) during implantation. **(A,B)** Right anterior oblique (RAO) and left anterior oblique (LAO) fluoroscopic images showed the sites of LBBP pacing lead. **(C,D)** Baseline QRS duration (QRSd) and paced QRSd upon pacing left bundle branch. **(E,F)** Sti-left ventricular activation time (LVAT) at low and high outputs.

All 34 patients successfully underwent CRT using LBBP (Figures 1A,B). An ECG showed that QRSd significantly decreased upon pacing the left bundle branch (Figures 1C,D). As shown in Table 2 and Supplementary Figure S1A, QRSd narrows dramatically from 153.2 ± 1.7 ms at baseline to 111.9 ± 2.6 ms during 1-month of follow-up and then stays stably narrow at 6 months (107.8 ± 2.4 ms) and 12 months (104.7 ± 3.4 ms, all *p* < 0.01). The sti-LVAT remained the same at both low and high outputs when LBB was captured (Figures 1E,F). The average sti-LVAT was 80.4 ± 3.1 ms after LBBP perforation. The mean unipolar LBB capture threshold was 0.73 ± 0.21 V @ 0.4 ms (@ = at) upon the time of implantation and decreased and remained stable at 1 month (0.61 ± 0.32 V @ 0.4 ms), 6 months (0.56 ± 0.12 V @ 0.4 ms), and 12 months (0.62 ± 0.13 V @ 0.4 ms). The R-wave amplitudes were 8.58 ± 4.09, 8.93 ± 3.62, 11.40 ± 3.50, and 12.02 ± 5.68 mV at implantation, 1, 6, and 12 months. Unipolar pacing impedance was decreased rapidly over the first-month post-implantation and thereafter remained steady during follow-up (Table 2).

**Table 2:** Electrophysiological and pacing parameters.

	**At implant**	**At 1 months**	**At 6 months**	**At 12 months**
QRSd (ms)	153.2 ± 1.7	111.9 ± 2.6	107.8 ± 2.4	104.7 ± 3.4
Pacing threshold (V @ 0.4 ms)	0.73 ± 0.21	0.61 ± 0.32	0.56 ± 0.12	0.62 ± 0.13
R-wave amplitude (mV)	8.58 ± 4.09	8.93 ± 3.62	11.40 ± 3.50	12.02 ± 5.68
Impedance (Ω)	612.90 ± 156.74	458.00 ± 110.59	400.00 ± 46.34	475.25 ± 77.15

### NYHA functional class and echocardiographic parameters

**Figure 2:**
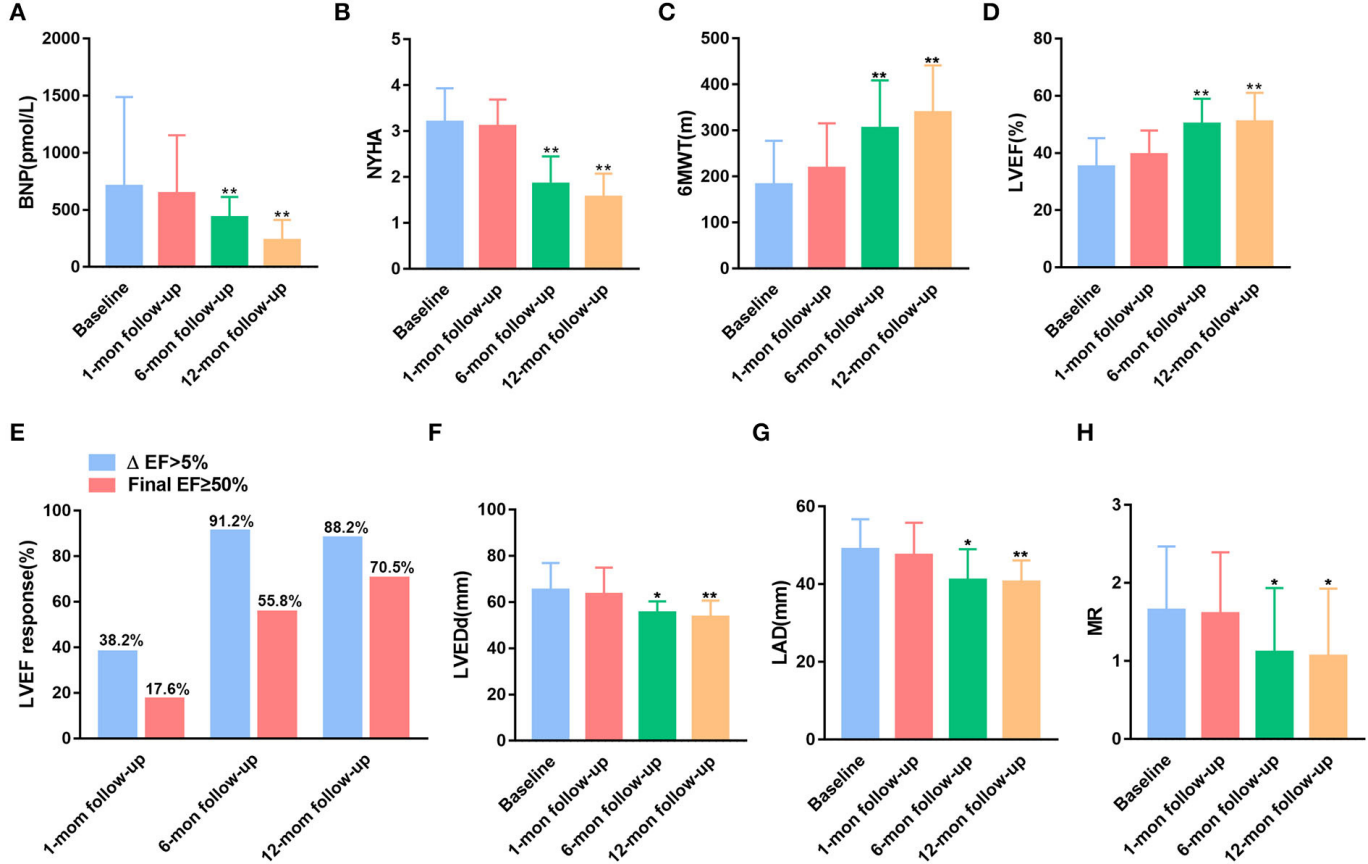
Comparisons of the New York Heart Association (NYHA) functional class and echocardiographic parameters at baseline and during the follow-up period. **(A)** Brain natriuretic peptide (BNP). **(B)** The NYHA classification. **(C)** 6-Minute walk test (6MWT). **(D)** Left ventricular ejection fraction (LVEF). **(E)** LVEF response rate. **(F)** Left ventricular end-diastolic dimension (LVEDd). **(G)** Left atrial dimension (LAD). **(H)** Mitral regurgitation (MR). *p < 0.05, and **p < 0.01 with baseline.

As shown in Figure 2A, compared with baseline BNP, patients with LBBP show no significant change in BNP at 1-month follow-up, whereas they have a significantly lower BNP at 6- and 12-month follow-up. Consistent with BNP, clinical heart function concerning NYHA and 6MWT was demonstrated to be improved during a follow-up period of 6 and 12 months (Figures 2B,C). LVEF was improved from a mean value 35.28 ± 1.70% at baseline to 50.26 ± 1.51% on follow-up of 6 months (*p* < 0.01) and increased to 51.09 ± 1.71% at 12 months (*p* < 0.01) after LBBP implantation (Figure 2D). An LVEF improvement >5% from baseline was defined as an LBBP response, and LVEF ≥ 50% was considered as a super-response. As shown in Figure 2E, 55.8 and 70.5% of patients have normalized LVEF (LVEF ≥ 50%) at 6 months and 12 months (Figure 2E). As shown in Supplementary Figure S1B, the mean change of LVEF in the general population is 2.44 ± 1.96% at 1-month follow-up, 11.47 ± 5.03% at 6-month follow-up, and 11.89 ± 5.05% at 12-month follow-up (both *p* < 0.01 with 1-month follow-up). For super-responders, the average change of LVEF was 2.44 ± 1.89% at 1-month follow-up, 11.44 ± 3.37% at 6-month follow-up, and 13.89 ± 2.18% at 12-month follow-up (both *p* < 0.01 with 1-month follow-up). The means of LVEDd and LAD were significantly lower at 6 months (65.3 ± 1.99 vs. 55.57 ± 1.81 mm and 49.03 ± 1.32 vs. 41.14 ± 2.98 mm, both *p* < 0.05) and 12 months (65.3 ± 1.99 vs. 53.58 ± 2.07 mm and 49.03 ± 1.32 vs. 40.67 ± 1.58 mm, both *p* < 0.01) of follow-up, whereas there was no non-significant reduction in LVEDd and LAD after 1-month LBBP implantation (Figures 2F,G). In addition, MR and TR were shown to be ameliorated at 6 and 12 months follow-up (Figure 2H; Supplementary Figure S1C).

### Complications of LBBP

There were no major acute adverse effects during device implantation. No lead displacements and LV perforations were documented. An increase in LBBP capture threshold >1.0 V @ 0.4 ms was not observed in any of the patients. None of the patients presented infection, LV thrombosis, and stroke during the follow-up period. During the follow-up of 12 months, no deaths or heart failure hospitalizations were observed.

## Discussion

In this retrospective, single-center, and observational study, we explored the feasibility, effectivity, and safety of a novel pacing technique in heart failure patients with cardiac resynchronization indications by using LBBP. The major findings of the current work are as follows: (1) the success rate of LBBP was high in patients with heart failure and LBBB. (2) The QRSd was significantly reduced after LBBP implantation and kept narrow during the follow-up period. (3) Obvious improvements in clinical heart function and echocardiographic response were found in CRT implantation *via* LBBP. (4) LBBP showed low and stable pacing thresholds with long-term follow-up.

A previous study showed that CRT *via* BVP was a traditional strategy to ameliorate prognosis and decrease mortality of chronic patients with heart failure (16). However, the anatomy of the coronary sinus differs from an individual, which contributed to difficulties in placing LV lead into the optimal vein branches. Owing to the anatomical features of the left bundle branch that has fasciculus widely under the endocardium of the left side of the interventricular septum, pacing the left bundle branch is easy by screwing the pacing lead helix through the interventricular septum to the left ventricular subendocardium. LBBP operation was successfully achieved in all 34 heart failure cases, which revealed that LBBP had a high implant success rate and was feasible at implant.

Cardiac resynchronization therapy is recommended for symptomatic patients with heart failure in sinus rhythm with a QRSd ≥150 ms and LBBB QRS morphology and with LVEF ≤ 35% despite optimal medical treatment in order to improve symptoms and reduce morbidity and mortality (3). Compared with BVP and HBP, LBBP was demonstrated to be easier to operate and improve LVEF with a low and stable threshold. Our data showed that LBBP pacing thresholds were 0.73 ± 0.21 V @ 0.4 ms at implant and then slightly decreased and remained stable and low (under 1 V @ 0.4 ms) during follow-up. Meanwhile, the mean QRSd had shortened by ~42 ms with LBBP operation for 1 month and was kept narrow in 12 months of follow-up. It has been reported that a narrower QRSd could lead to better mechanical synchronization of the ventricle (17). Pacing distal to the site of LBBB could correct LBBB and restore normal physiological left ventricular activation, which resulted in QRSd reduction (18). Thus, our study suggested that effective electrical and mechanical resynchronization was obtained from CRT through LBBP.

After 1 month of operation, nearly 38.2% of patients had a 5% increase of LVEF from baseline, and only 17.6% of patients had normalized LVEF. With the extension of follow-up, a significant increase of LVEF was observed in these heart failure patients with LBBB requiring CRT by LBBP. Super-response to LBBP implantation was achieved in 55.8 and 70.5% of patients after 6- and 12-month follow-up, respectively. The results were similar to the project conducted by Huang et al. (19), in which 75% of the non-ischemic population had normalized LVEF (≥50%) at 1 year by using LBBP. Apart from the high echocardiographic response, improved clinical manifestations were also achieved during long-term follow-up that included modified NYHA functional class, decreased BNP, and increased 6MWT. These results suggested that cardiac systolic function was improved during long-range follow-up visits of LBBP implantation.

Though LBBP was demonstrated to deliver effective cardiac resynchronization by correcting LBBB, pacing the left bundle branch did not allow normal physiological activation of both ventricles due to delayed LV lateral wall activation. However, we found that QRSd was decreased and maintained normal after LBBP implantation, and ventricular function did not deteriorate during follow-up, the results of our study might lie in heart failure patients with typical LBBB meeting Strauss criteria and the majority had dilated cardiomyopathy and were included in the present work. In addition, no adverse operative-related complications occurred in the process of implantation and follow-up, which revealed that LBBP was a safe approach to physiological pacing.

In conclusion, we identified that LBBP is a rational method of physiological pacing in heart failure patients with LBBB, as it leads to improvements in ventricular structure and function. Low and stable capture thresholds are associated with LBBP. However, the present work was conducted in a single-center with a small cohort. Large-scale, long-term, and randomized controlled clinical trials remain to be done to further estimate the clinical advantages and safety of LBBP in comparison with BVP and HBP in CRT candidates.

## Data availability statement

The raw data supporting the conclusions of this article will be made available by the authors, without undue reservation.

## Ethics statement

The studies involving human participants were reviewed and approved by the Ethics Committee of Jinling Hospital Affiliated to Nanjing University School of Medicine. The patients/participants provided their written informed consent to participate in this study.

## Author contributions

JL and LW conceived and designed the experiment. YG, YL, and YZ analyze the data. XL and TT performed the statistical analysis. JL and YG wrote the manuscript. JL, QZ, and JG revised the manuscript. All authors contributed to the article and approved the submitted version.

## Conflict of interest

The authors declare that the research was conducted in the absence of any commercial or financial relationships that could be construed as a potential conflict of interest.

## Publisher's note

All claims expressed in this article are solely those of the authors and do not necessarily represent those of their affiliated organizations, or those of the publisher, the editors and the reviewers. Any product that may be evaluated in this article, or claim that may be made by its manufacturer, is not guaranteed or endorsed by the publisher.

## Supplementary material

Supplementary Figure S1 Comparisons of QRS duration (QRSd) and TR at implantation and during follow-up. **(A)** The intrinsic and paced QRSd during left bundle branch pacing (LBBP). **(B)** The change of left ventricular ejection fraction (LVEF) in the general population and super-responders. **(C)** Mitral regurgitation (MR) at baseline and follow-up. ^*^*p* < 0.05, and ^**^*p* < 0.01 with baseline.
