# Supplementary figures and images for: Cardiac resynchronization therapy in heart failure patients by using left bundle branch pacing
Source: Front Cardiovasc Med. 2022 Aug 23;9:990016. doi: 10.3389/fcvm.2022.990016 (PMC9445246; doi:10.3389/fcvm.2022.990016)

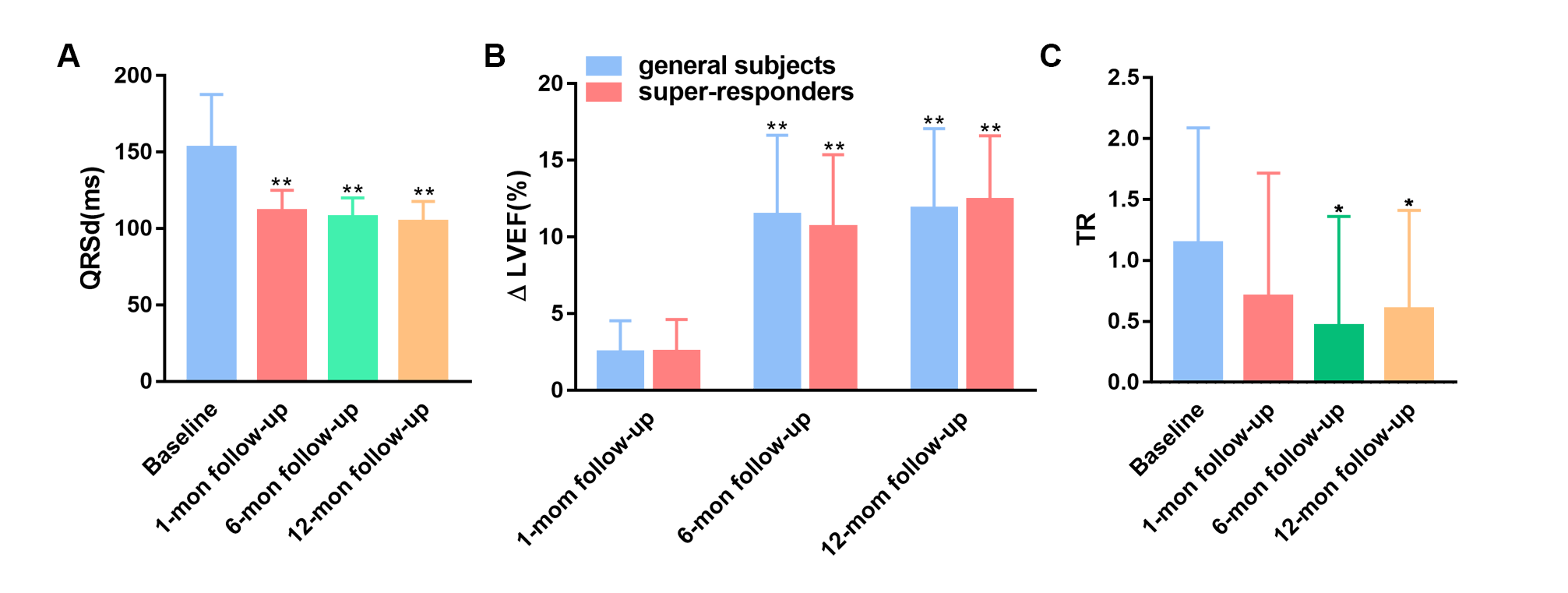

Supplement: Supplementary Figure S1 — Comparisons of QRS duration (QRSd) and TR at implantation and during follow-up. (A) The intrinsic and paced QRSd during left bundle branch pacing (LBBP). (B) The change of left ventricular ejection fraction (LVEF) in the general population and super-responders. (C) Mitral regurgitation (MR) at baseline and follow-up. *p < 0.05, and **p < 0.01 with baseline. [file Image_1.TIF]
